# Evaluation of Treatment Outcomes in Patients with Stiff Person Syndrome with Rituximab vs. Standard of Care

**DOI:** 10.7759/cureus.1387

**Published:** 2017-06-24

**Authors:** Stephen Rineer, Timothy Fretwell

**Affiliations:** 1 University of Central Florida, UCF College of Medicine

**Keywords:** stiff person syndrome, rituximab

## Abstract

Stiff Person Syndrome (SPS) is a rare neurological disorder that primarily affects the ability to relax musculature. This results in affected muscle groups remaining in constant contracture, leading to painful spasms that have significant morbidity and impact the patient's quality of life. Disease prevalence is one to two persons in a million, and as a result, very few randomized controlled studies have examined the efficacy of various treatment regimens. One notable study examined intravenous immunoglobulin (IVIG) and its efficacy in the treatment of SPS. This study found that using IVIG was of significant benefit in improving stiffness in SPS. However, beyond this, immune modulating therapy is limited by lack of peer-reviewed evidence. The use of rituximab has been reported in cases of SPS that are refractory to treatment with IVIG and has had mixed outcomes. Our search of the literature involved examining case reports of patients with diagnosed SPS, who had been initially treated with the standard of care and were then placed on treatment with rituximab. Our review of the available case reports demonstrates an increase in SPS remission correlating with the frequency of dose. However, the limited number of case reports available limits conclusions related to the treatment of SPS. More studies are needed to assist in guiding therapy for SPS.

## Introduction and background

Stiff Person Syndrome (SPS) is a rare neurological movement disorder that occurs at the rate of one to two persons per million. The hypothesized mechanism of SPS is a dysregulation of neuronal firing, leading to sustained muscular contraction, stiffness, and rigidity. However, the details of the exact pathogenic mechanism has yet to be elucidated. Evidence exists that implicates an underlying autoimmune process potentially involving both the B-cell and T-cell mechanisms. The autoimmune process is further supported by the occurence of other autoimmune conditions along with SPS. Examples of this include diabetes mellitus, hypothyroidism, pernicious anemia, and vitiligo [1]. There is also evidence of a paraneoplastic variant of SPS.

The autoimmune nature of SPS has been illustrated by the presence of anti-glutamic acid decarboxylase (anti-GAD) antibodies and/or anti-amphiphysin antibodies in the serum of patients with SPS. High titers of anti-GAD are highly specific for the disease. However, up to 30% of patients with SPS are negative for anti-GAD antibodies. It is thought that these antibodies interfere with the inhibitory effects of gamma-aminobutyric acid (GABA) in the central nervous system (CNS), leading to constant muscle contractions [1]. Antibodies of the post-synaptic GABA-A receptor have also been found in approximately 70% of patients with SPS, which may also have pathogenic implications. A T-cell-based mechanism has been considered becaise of the 30% rate of co-occurrence of type 1 diabetes mellitus (T1DM) in patients with SPS. It is thought that microglia could become sensitized to a form of glutamic acid decarboxylase (GAD) protein and then present the protein to T-cells, leading to neural damage via a T-cell mediated mechanism [2]. This is supported by the understood role of T-cells in the pathogenesis of T1DM and the documented rates of T1DM and other autoimmune conditions in SPS patients.

With a pathogenesis that is poorly understood, the management options available for SPS mainly involve symptomatic treatment with baclofen, diazepam, and other similar neuromuscular drugs that work to reduce muscle rigidity and spasms. The argument for the autoimmune basis of the disease is further strengthened by the documented effectiveness of various first-line treatments in other autoimmune conditions, including intravenous immunoglobulin (IVIG), steroids, and plasmapheresis [3]. Another treatment option that has been explored in SPS case reports is rituximab because of its ability to target and destroy B-cells. Rituximab binds to the CD20 antigen on mature B-cells, leading to B-cell lysis, while sparing the precursor B-cells. Rituximab has been shown to be useful in delaying the progression of disease in other neurological autoimmune disorders and may show a similar benefit in SPS [4]. This review seeks to examine the reported efficacy of rituximab in treating SPS when it is refractory to other forms of treatment.

## Review

To be included in this review, the patients in the case report literature had to have a clinical presentation consistent with a diagnosis of SPS and a positive serology (anti-GAD or anti-amphiphysin) or an electromyography (EMG) consistent with SPS (as seen in Table 1). The outcomes of the treatments involved were evaluated based on EMG changes, anti-GAD titers, and clinical mobility. Table 1 references each of the nine cases of rituximab use in patients with reported SPS.

**Table 1:** Chart with overview of cases

*#*	Age	Sex	Serology	EMG	Current Medications	Type of SPS	Rituximab Dosage	Outcome
*1*	41	F	Anti-GAD	Spontaneous involuntary motor unit potentials	Baclofen, dantrolene, fentanyl, diazepam, diamorphine	Classic SPS [5].	One 375 mg/m^2^ dose followed by four doses given weekly after six weeks	Improved symptoms and mobility, lasting clinical remission
*2*	53	M	Anti-GAD Anti-amphiphysin	Spontaneous involuntary motor unit potentials affecting both agonist and antagonist muscles	IVIG, diazepam, baclofen	Classic SPS [6].	Four 375 mg/m^2^ doses given weekly followed by a single 375 mg/m^2^ dose at eight months	Complete sustained remission
*3*	59	F	Anti-GAD	Spontaneous motor unit activities	IVIG, plasmapheresis, diazepam, baclofen, alprazolam	Partial SPS [7].	Three 375 mg/m^2 ^doses, initial dose with following doses at three and four months	Clinical remission and good neurological condition at 23 months
*4*	41	F	Anti-GAD	Continuous motor activity	Diazepam, botulinum toxin	Classic SPS [8].	Two 375 mg/m^2^ doses, 15 days apart	Clinical improvement with increasing Anti-GAD titers
*5*	39	F	Anti-GAD	Not available	Diazepam	Classic SPS [9].	Two 375 mg/m^2^ doses, seven days apart	Remission followed by relapse
*6*	12	M	Anti-GAD	Not available	Levetiracetam, diazepam, clonazepam, IVIG, baclofen, gabapentin	Classic SPS [10].	Two 500 mg/m^2^doses, 14 days apart	Remission and improved ambulation
*7*	34	M	Anti-GAD	Continuous motor activity	Diazepam, IVIG	Classic SPS [11].	Four 1000 mg doses, two doses at 18 weeks, two doses at 54 weeks, each 14 days apart	No profound benefit seen
*8*	34	M	Anti-GAD	Continuous motor activity	Diazepam, IVIG	Classic SPS [11].	Four 1000 mg doses, two doses at week zero, two doses at 54 weeks, each 14 days apart	No profound benefit seen
*9*	56	M	Anti-GAD	Not available	Levetiracetam, gabapentin, diazepam, IVIG, steroids	Classic SPS [12].	Four 375 mg/m^2 ^doses, over 3 months	Complete sustained remission 1-year post-therapy

Of the case reports that were reviewed, clinical improvement was seen in seven of the nine patients. However, the dose and schedule of rituximab varied among those patients. Patients 1 through 5 and 9 received 375 mg/m^2 ^with variable dosing intervals and total number of doses. Remission was seen with fewer doses in several patients. It is important to note that clinical improvement was seen in all six patients, though some required an additional dose because of the recurrence of symptoms several months after the original dose. Patient 5 did relapse in her disease after experiencing remission; however, it is important to note that she received the fewest number of doses of the patients who received 375 mg/m^2 ^doses of rituximab. No comment was made with reference to repeat dosing following a relapse or after the initial duration of remission. Patient 6 received two doses of 500 mg/m^2^ 14 days apart and clinical improvement was observed despite the difference in dose size and frequency compared with the patients mentioned earlier. Finally, in a double-blind crossover study, a 1,000 mg dose of rituximab was given to a monozygotic set of twins with SPS. This study gave doses at intervals of 2 weeks and compared with the use of saline. These patients were given a follow-up two-week treatment at 54 weeks of 1,000 mg. No clear benefit was shown upon functional analysis; however, the authors suggested that it may have prevented a worsening of the disease.

Dosages of rituximab show a trend toward increased doses being effective in this limited population size. Patients 1 and 2 received five doses of rituximab, with four given over four weeks, and they demonstrated lasting clinical improvement. Patient 9 received four doses of rituximab over the course of three months, prompting lasting remission that was still present one year later. The double-blind crossover study with Patients 7 and 8 also reported four doses; however, these were spaced out over the course of one year and very little clinical improvement was observed. Patient 3 was administered three doses, with one initial dose and two more doses three months later, with a month separating the final two doses. This patient remained in “good neurologic condition at 23 months” following the rituximab treatment per the case report [7]. Patient 4 was administered rituximab 15 days apart and showed clinical improvement. Patient 6 received two doses 14 days apart with sustained improvement; however, the paper did not list a duration of improvement. Patient 5 was given two doses of rituximab seven days apart and improvement was initially seen but followed by relapse. It is important to note that Patient 5 was complicated by benzodiazepine withdrawal syndrome.

Based on this review of the dosing schedule and quantity of rituximab administered, it would seem that a treatment plan involving frequent doses of 375 mg/m^2 ^over a shorter period of time was the most-effective treatment for SPS. To draw any conclusions, the limited sample size warrants more research in this area.

## Conclusions

At this time, there is limited evidence for proving the effectiveness of rituximab use in SPS. The case-based evidence presented in this literature review includes anti-GAD positive patients who have failed symptomatic therapy. No cases have been reported of rituximab use in seronegative or paraneoplastic etiologies. A phase II clinical trial conducted by the National Institute of Health was recently completed and may help to further delineate the role of rituximab in SPS. Further, several of these case reports failed to define or quantify complete remission (i.e., no more pain, regained muscle function, lack of muscle spasms, and so on). A definition of remission and a means of quantifying treatment response is necessary to not only monitor outcomes but also to determine the usefulness of various treatments relative to each other. Additionally, because of the various pathogenic mechanisms that may be involved in SPS, criteria that may make rituximab a first-line treatment for selected patients with SPS need to be determined. For example, a patient with a T-cell based mechanism for their disease pathogenesis would be a poor candidate for rituximab treatment given the confounding disease process when compared to the mechanism of action of rituximab. The varied nature of SPS pathogenesis may be the enigma behind the differing responses to treatment not only with rituximab, but also with IVIG and other treatment modalities. 
